# 

**Published:** 1895-03

**Authors:** 


					﻿THE
Southern Medical Record.
A Monthly Journal of Medicine and Surgery.
Vol. XXV.	ATLANTA, GA., MARCH, 1895.	No. 3.
EDITORS:
1. W. GRIGGS, M. D.	WILLIS F. WESTMORELAND, M. D.
I. McFADDEN GASTON, M. D.	LOUIS H. JONES, M. D.
DAN. H. HOWELL, M. D., Business Manager.
WM. S. GOLDSMITH, M. D., Managing Editor.
TERMS: $2.00 Per Annum; Single Copy, 20 Cents.	Contents on Page 0.
Entered at the Post-office at Atlanta, Ga., as Second-Class Matter.
The Foote & Davies Co., Atlanta, Ga.
				

